# Transcranial Direct Current Stimulation of the Dorsolateral Prefrontal Cortex Modulates Repetition Suppression to Unfamiliar Faces: An ERP Study

**DOI:** 10.1371/journal.pone.0081721

**Published:** 2013-12-04

**Authors:** Marc Philippe Lafontaine, Hugo Théoret, Frédéric Gosselin, Sarah Lippé

**Affiliations:** 1 Department of Psychology, Université de Montréal, Montréal, Canada; 2 Sainte-Justine University Hospital Research Center, Montréal, Canada; Tel Aviv University, Israel

## Abstract

Repeated visual processing of an unfamiliar face suppresses neural activity in face-specific areas of the occipito-temporal cortex. This "repetition suppression" (RS) is a primitive mechanism involved in learning of unfamiliar faces, which can be detected through amplitude reduction of the N170 event-related potential (ERP). The dorsolateral prefrontal cortex (DLPFC) exerts top-down influence on early visual processing. However, its contribution to N170 RS and learning of unfamiliar faces remains unclear. Transcranial direct current stimulation (tDCS) transiently increases or decreases cortical excitability, as a function of polarity. We hypothesized that DLPFC excitability modulation by tDCS would cause polarity-dependent modulations of N170 RS during encoding of unfamiliar faces. tDCS-induced N170 RS enhancement would improve long-term recognition reaction time (RT) and/or accuracy rates, whereas N170 RS impairment would compromise recognition ability. Participants underwent three tDCS conditions in random order at ∼72 hour intervals: right anodal/left cathodal, right cathodal/left anodal and sham. Immediately following tDCS conditions, an EEG was recorded during encoding of unfamiliar faces for assessment of P100 and N170 visual ERPs. The P3a component was analyzed to detect prefrontal function modulation. Recognition tasks were administered ∼72 hours following encoding. Results indicate the right anodal/left cathodal condition facilitated N170 RS and induced larger P3a amplitudes, leading to faster recognition RT. Conversely, the right cathodal/left anodal condition caused N170 amplitude and RTs to increase, and a delay in P3a latency. These data demonstrate that DLPFC excitability modulation can influence early visual encoding of unfamiliar faces, highlighting the importance of DLPFC in basic learning mechanisms.

## Introduction

Repetition of a visually presented stimulus leads to suppression of neural activity in the cortical areas responsible for processing the stimulus [1], [2]. Much evidence over recent years has been reported for this phenomenon, known as repetition suppression (RS) [3], across a broad range of neuroimaging methods, cortical regions, neural scales and study protocols (for a review, see Grill-Spector et al. [4]). Behaviourally, RS has been associated with perceptual priming, a basic form of memory formation and learning whereby accuracy and reaction time (RT) are implicitly improved in recognizing a previously presented stimulus [1], [5], [6]. Both RS and priming are incremental in nature, enhancing their effects as a function of the number of presentations of a stimulus [2], [7]. Also, duration of stimulus presentation at encoding influences RS and priming at retrieval in almost identical patterns [8].

RS is a fundamental sensory process, but is also embedded in declarative memory and higher associative forms of learning. For instance, stronger RS in the temporal cortex at retrieval, as detected by blood-oxygen level dependent (BOLD) signal reduction, is associated with higher perceived familiarity for previously encoded visual stimuli, which may provide a basis for declarative memory formation [9]. In clinical populations, compromised neural RS is often linked to impairments in the capacity to learn new information visually, including unfamiliar faces. In elderly patients with Alzheimer’s disease, poor performance in recognition tests assessing episodic memory for faces and corresponding names is associated with reduced suppression of activity at encoding in medial temporal lobe structures. This contrasts with cognitively unimpaired young and elderly subjects showing stronger RS effects and normal retrieval performance [10]. In acquired prosopagnosia, the inability to recognize individual faces has been associated with a lack of RS to repeated identical faces in the right fusiform gyrus compared to control subjects [11]. In addition, learning of novel faces is severely impaired in the absence of RS to unfamiliar faces in the fusiform face area (FFA) as characterized in developmental prosopamnesia [12].

These neuropsychological signs suggest that the capacity to learn new faces is highly dependent on the presence of RS in face-specific cortical areas. Investigations into the RS properties of the N170 event-related potential (ERP) component in normal subjects provide further insight into the reason why. The N170 component is a robust finding in ERP research and is described as a negative deflection peaking over occipito-temporal sites around 170ms post-stimulus, maximal for face stimuli and thought to reflect early structural detection and perceptual processing of faces [13], [14]. RS is reflected in variations of N170 peak amplitude and latency values in response to repeated faces relative to novel ones. Although RS effects on latency are inconsistent, N170 amplitude is usually reduced in a way similar to reduction of BOLD activity in the FFA and both markers may reflect a neural mechanism for the early stages of face identity learning [15]–[17]. Indeed, in tasks demanding explicit encoding of individual faces by repetition, episodic memory performance and N170 RS are equal for upright and inverted faces [18], [19]. However, N170 RS is specific to upright faces in a task where attention is driven away from face identity [20]. Given that upright faces can be processed implicitly as opposed to inverted faces which require attention [21], repeated processing of individual face identities is thereby isolated in inducing N170 RS. Further evidence where N170 reduces exclusively to repetitions of a same unfamiliar face identity has been reported [16], [22]–[25]. Thus, because there can be no learning of new face identities without prior processing, N170 RS is assuredly a mechanism involved in rendering unfamiliar faces familiar. Still, the underlying neural model that would explain or give rise to RS and face encoding is unclear.

The dorsolateral prefrontal cortex (DLPFC) is a source of top-down control that has been shown to influence the course of bottom-up visual processing through increases in extrastriate neural activity enhancing attention to elements of the visual field [26], [27]. In patients with DLPFC damage, this positive influence on visual processing is compromised, leading to diminished ability in discriminating visual stimuli in the hemifield contralateral to lesions and to ERP perturbations over occipito-temporal sites in the ipsi-lesional hemisphere [28]. Results from studies using long-range functional connectivity analysis of fMRI data [29], [30] support the presence of a link between prefrontal top-down control and modulations in visual processing and behaviour. In support of a causal link, right prefrontal activity disruption by transcranial magnetic stimulation (TMS) during encoding of visually presented non-verbal items entails a decrease of early sensory responses in occipito-temporal cortex as well as working memory impairment [31]. As proposed by the HERA model [32], [33], prefrontal regions also generally contribute to memory formation and right DLPFC activation may specifically predict successful encoding of faces [34], [35]. Face identity learning therefore depends not only on RS within face-specific areas, but also on proper dynamic interactions between occipito-temporal cortex and prefrontal areas [36].

Although many neural models for RS have been put forth [4], one that involves the contribution of top-down control has shown promise in accounting for both neural and behavioural effects. Indeed, based partly on results indicating that RS occurs jointly with increases in cortico-cortical connectivity [37] and that activity in prefrontal cortex ostensibly precedes RS in temporal lobe structures [38], some have proposed a theoretical model where RS in early processing areas is a consequence of the progressive reduction in prediction error from higher-order, associative areas of cortex, the DLPFC amongst them [39], [1]. As the difference between bottom-up sensory input and experience-dependent, top-down prediction is lessened through repetition, visual processing becomes more efficient and may lead to the reduction of activity needed to represent the stimulus and the enhanced ability to recognize and retrieve items from memory. Supporting this model, in studies where the probability of repetition of faces is manipulated, higher probability of repetition entails stronger fMRI adaptation or RS effects in the FFA [40] and earlier areas as well [41]. In other words, stimuli with higher repetition probability become expected by higher cortical areas; this reduces their prediction error and enhances RS relative to stimuli with lower repetition probability. Of relevance to the present study, it has recently been proposed that this top-down model may be preferential for facial stimuli, as repetition probability of everyday objects has no effect on RS strength [42]. In ERP studies, the P300 and its frontal subcomponent P3a provide further illustration of this model. The P3a is thought to reflect the detection of novel stimuli to which attentional resources are allocated, regardless of sensory modality [43], [44]. Frontal lobe and DLPFC patients are generally impaired in novelty detection and show reduced P3a amplitude [45], [46]. However, augmented P3a amplitude can reflect higher attention, which enhances encoding of a stimulus [44]. Furthermore, with repeated presentation of a novel stimulus, P3a amplitude suppresses as memory for this stimulus is formed [47]. This RS of the P3a is dependent on the integrity of a circuit involving the DLPFC and occipito-temporal areas. Indeed, P3a RS is absent in epileptic patients having undergone medial temporal lobe resection [48]. In this case, without proper processing of a stimulus in occipito-temporal regions, the DLPFC would have no representation with which to compare its predictions, and encoding may become impaired. Thus, in light of its sensitivity to top-down contributions in learning mechanisms involving occipito-temporal areas, the present study uses P3a to evaluate changes in DLPFC function caused by noninvasive brain stimulation during learning of unfamiliar faces.

Transcranial direct current stimulation (tDCS) is a non-invasive form of electrical brain stimulation which allows transient and superficial cortical excitability modulation through application of a weak electrical current via electrodes placed on the surface of the scalp. Cortical excitability modulation is polarity-dependent: anodal stimulation increases excitability of the underlying cortex whereas cathodal stimulation decreases it [49]. Unlike TMS, tDCS does not induce action potentials but modulates spontaneous firing rates and, when applied for a sufficient amount of time (∼10 minutes), induces long-lasting plasticity changes through NMDA receptor efficacy modulation [50]–[52]. Although the increased use of tDCS in recent years began in the context of application over the motor cortex, an increasing amount of literature now describes its effects on higher cognitive functions such as planning abilities [53], risk-taking and decision-making [54]–[56], working memory [57] and learning [58], [59]. Of note, polarity reversal between active stimulation conditions can yield differential effects on cognitive function including risk-taking [56] and learning [60].

Based on the body of evidence reviewed, we hypothesized that application of tDCS over the DLPFC would modulate N170 RS over occipito-temporal sites in response to repeated presentation of unfamiliar faces. Differential effects would arise in a polarity-dependent fashion whereby anodal tDCS would have an effect opposite to that induced by cathodal stimulation. Similarly, the P3a component would also show polarity-dependent changes in RS and amplitude, reflecting top-down influences including attention. As tDCS-induced cortical excitability modulation is transient [49], we expected that observed effects on ERPs would decay over time. Behavioural measures should follow patterns similar to RS modulation, which is to say enhanced RS would be associated with better accuracy and/or shorter RT in a subsequent assessment of memory, and the opposite for RS impairment.

## Materials and Methods

### Ethics statement

**Figure 1:**
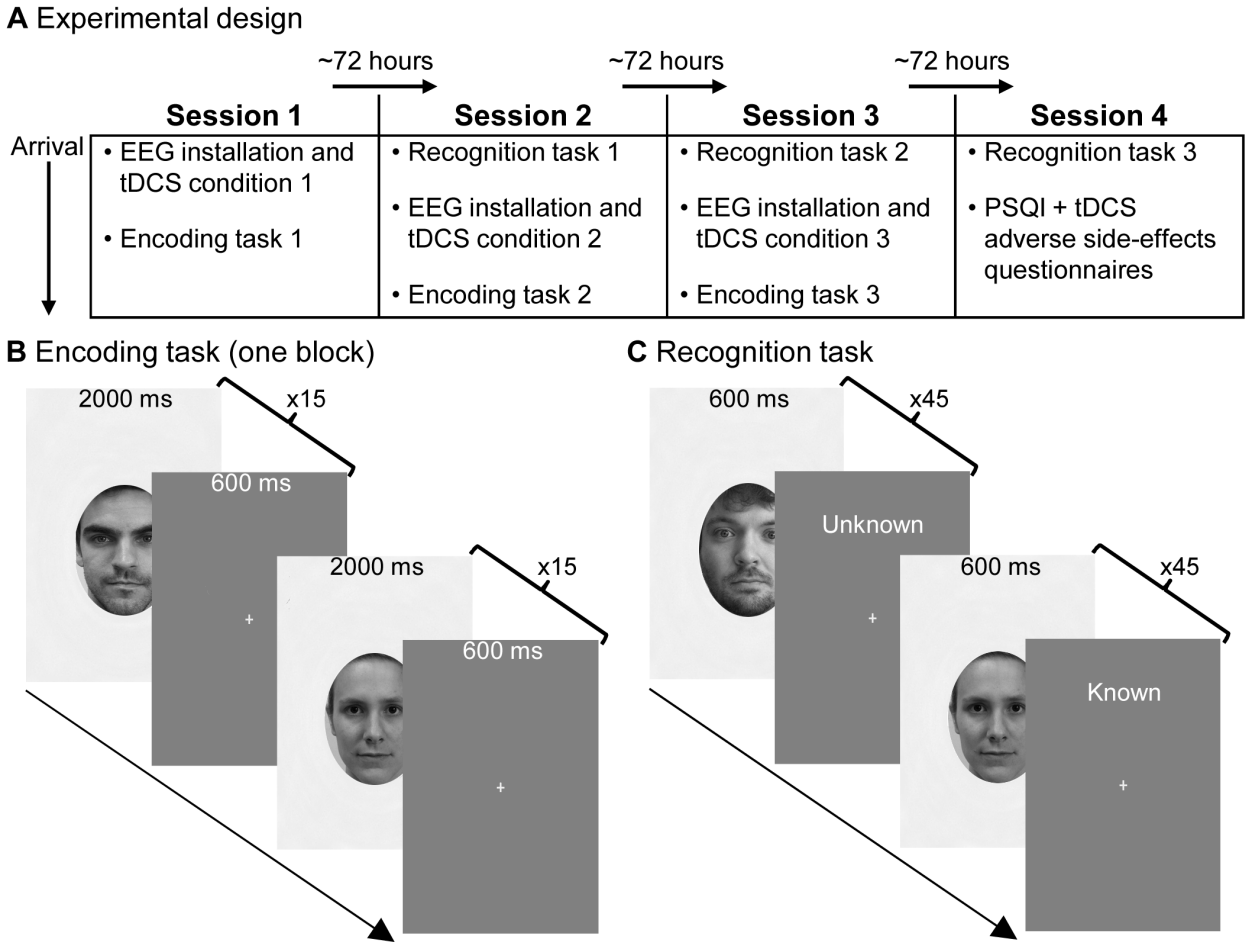
Experimental design and tasks. (A) tDCS conditions and tasks were independently randomly ordered across participants. Encoding and recognition, as well as tDCS conditions were separated by ∼72 hours to test long-term recognition and avoid cross-over cortical excitability modulation effects. (B) Example of one encoding task block. Encoding tasks consisted of three blocks, each comprising 15 different unfamiliar faces presented 15 times consecutively. (C) Example of a corresponding recognition task. The 45 unfamiliar faces encoded ∼72 hours earlier are presented amongst 45 novel faces. For each face, participants responded either "known" or "unknown" (here, correct answers are indicated on the intertrial interval). Stimuli in this figure are not the originals, but similar stimuli used for illustrative purposes only.

The project was reviewed and approved by the *Comité d’éthique de la recherche de la Faculté des arts et des sciences* (*CÉRFAS*) of the University of Montreal and was conform to the Declaration of Helsinki. All participants gave written informed consent prior to testing. Subjects in Figure 1B, C have given written informed consent, as outlined in the PLOS consent form, to publication of their photograph.

### Participants

Participants were 14 healthy young adults (8 males and 6 females, range: 21–31 years; mean ± SD: 23.5±2.37 years), all undergraduate and graduate students of the University of Montreal. All participants had normal or corrected-to-normal vision and reported no history of neurological or psychiatric disorders. Behavioural and ERP data of two participants were rejected because of concerns they did not perform the encoding task correctly (recognition accuracy ≤50%). An additional participant’s EEG data were rejected because of excessive artifacts. Behavioural data of this participant were also removed from analyses, to maximize comparability of results between behavioural and EEG analyses. Behavioural and EEG analyses therefore included 11 participants. These remaining participants were right-handed.

### Stimuli

Stimuli were 270 different unfamiliar male (177) and female (93) grayscale Caucasian faces. Images were selected from the FERET database on the criteria of their neutral expressions and absence of glasses, earrings or beards, to avoid any facilitation in learning or recognition based on these features. As this study aimed at characterizing the contribution of top-down processes in basic learning, it was essential to minimize any possible confounds arising from bottom-up processes by controlling for low-level image properties such as size, spatial location, luminance, contrast and spatial frequency. Firstly, to equate size and spatial location across stimuli, we aligned the internal attributes of faces using Matlab functions (available at http://www.mapageweb.umontreal.ca/gosselif/alignTools/) and a procedure similar to that described by Taschereau-Dumouchel and colleagues [61]. Broadly, images were rotated, translated and scaled such that the eyes and mouths were aligned with the average coordinates of these attributes calculated from a total of 12 annotations subjectively placed on each stimulus in the set. Secondly, a model (252×323 pixels) ellipse mask was placed around the faces, the exterior of which was cropped including the hairline and ears, leaving only the internal attributes of the face against a white background. Finally, luminance (13.3 cd/m^2^), contrast and spatial frequency were equated across stimuli using functions from the SHINE toolbox for Matlab (available at http://www.mapageweb.umontreal.ca/gosselif/SHINE/), conceived by Willenbockel et al. [62] specifically for this purpose.

### Transcranial direct current stimulation

tDCS was applied using a Magstim DC stimulator via two rubber electrodes (one cathode, one anode) placed inside 35 cm^2^ sponges soaked in sodium chloride solution. Electrodes were installed on the scalp using a bifrontal configuration over positions F3 and F4 in accordance with the international 10/20 EEG electrode placement system. Using a repeated-measures design, participants were administered all three tDCS conditions: right (F4) anodal/left (F3) cathodal, right cathodal/left anodal and sham. For each active tDCS condition, the battery-driven stimulator was programmed to deliver a 1.5mA direct current for 15 minutes, with 10 seconds of linear fade in and fade out. These current intensity and stimulation duration parameters were chosen for their reported ability to limit undesirable side-effects while effectively inducing cortical excitability modulation [63], [64]. In the single-blind sham condition, electrodes were applied bifrontally and stimulation was maintained for the first 30 seconds only, a now standard method introduced by Gandiga, Hummel, & Cohen [65]. Although many tDCS electrode configurations were considered, the bifrontal type was chosen for its reliability in yielding behavioural results linked to high-level, executive function modulation [54]–[56]. Of note, bilaterally applied tDCS inherently allows sufficient distance between electrodes, thus reducing chances of shunting of the current through the skin or cerebrospinal fluid [66].

### Tasks and procedure

For each tDCS condition, an encoding task was administered, followed by a matching recognition task (see Figure 1A). Each encoding/recognition task used a different subset of randomly selected face stimuli and was matched with a tDCS condition. The order in which participants would undergo tDCS conditions and encoding/recognition tasks was randomized for each participant. For all tasks, participants were comfortably seated in a sound-attenuated, electrically shielded room, at 70 cm viewing distance from centrally displayed stimuli subtending 10.3×15.7° visual angle, presented using E-Prime 2.0 software (on a 19", 1280×1024 pixel resolution monitor).

**Encoding task (** **Figure 1B** **).** The EEG cap was installed first with placement of tDCS electrodes underneath, ensuring precision and consistency of installation across conditions and participants. However, to avoid any electrical contamination of EEG signals, tDCS was administered prior to the start of recording such that the two systems were not simultaneously active. During tDCS, participants were instructed to close their eyes and relax while remaining awake. Both EEG recording and the encoding task started immediately following removal of tDCS electrodes and verification of impedances (∼5 minute delay). Each encoding task comprised 3 separate blocks. In each block, 15 different face identities were presented consecutively 15 times each. Each encoding task therefore contained 45 different face identities presented 15 times each for a total of 675 (45×15) trials per encoding task. In later analyses, blocks were added as a factor to assess the expected decay of tDCS effects over time. Each encoding task block lasted 10 min 13 sec. Participants were not instructed to use any particular encoding strategy but were asked to attentively observe the faces on which they would subsequently be tested. Stimuli were presented for 2000ms with a 600ms intertrial interval showing a central fixation cross.

**Recognition task (** **Figure 1C** **).** The 45 encoded faces were presented amongst 45 novel distractor faces in 90 randomly ordered trials. In each trial, a face appeared for 600ms followed by a fixation cross at which point participants had to perform a forced choice old/new discrimination decision using the left mouse button for known (i.e.: previously encoded) faces and the right button for unknowns. Participants were instructed to respond as fast as possible without making mistakes. Reaction time (RT) and accuracy were recorded at every trial.

To assess the long-term repercussions of DLPFC activity modulation during encoding and avert any cross-over effects from one tDCS stimulation condition to the next, tasks and conditions were administered at three-day (∼72 hours) intervals. This led to the four-session experimental design depicted in Figure 1A, where the last session comprised a recognition task only and two questionnaires. Because sleep is known to support memory functions [67] and sleep disorders to cause memory formation impairments [68] and waking EEG abnormalities [69], participants were asked to complete the Pittsburgh Sleep Quality Index (PSQI) questionnaire [70] to control for sleep quality. In addition, to assess the variability and intensity of adverse side-effects caused by tDCS, participants also completed a questionnaire similar to that suggested by Brunoni et al. [63].

### Electrophysiology

During encoding tasks, continuous EEG was recorded from 32 Ag/AgCl sintered electrodes (10/20 system) mounted in a Quik-cap (Compumedics). Data were acquired at 500 Hz sampling rate and high-pass filtered at 0.1 Hz with NeuroScan 4.5. Linked mastoids were used as reference and impedances were kept below 5 kΩ. Vertical and horizontal eye movements were monitored by EOG with four additional bipolar electrodes positioned on the outer canthus of each eye as well as above and under the orbit of the left eye.

Offline signal processing and averaging was performed (using BrainVision Analyzer 2) separately for each condition, participant and channel. EEG raw data were first segmented into 675 trials lasting 2200ms each (200ms baseline) based on stimulus onset marker positions. A high-pass digital filter was applied at 0.5 Hz with an additional low-pass filter set to 50 Hz (24dB/octave) and 60 Hz notch filter. Eye movement artifacts were corrected by algorithm [71] and trials containing segments exceeding ±125 µV were discarded. Trial data were corrected relative to the -100ms pre-stimulus baseline. Finally, for every individual face identity, the first five, middle five and last five trials were averaged separately (later referred to as Av1, Av2 and Av3 trial averages) for subsequent ERP analysis. Although RS has been reported to happen as early as the second presentation of a stimulus in adults [72], [25], some accounts suggest it may persist at later trials [2] thus, partitioning of data into three separate averages was carried out to assess different stages of the RS process.

### Behavioural and ERP statistical analyses

All analyses were conducted using SPSS Statistics 20 software. Correct answers in the recognition tasks were defined as correct identification of known and novel faces. Accuracy was then calculated as percentages of correct answers for every participant and condition. RT and accuracy data were first entered in two-way repeated-measures ANOVAs with tDCS condition (3) and stimulus familiarity (previously encoded vs. novel distractors) as within-subjects factors. To assess the specificity of tDCS effects, RT and accuracy data were subsequently divided into those of faces encoded while under the influence of tDCS and those of novel faces (i.e.: first presented ∼72 hours following active tDCS). Encoded faces RTs and accuracy were entered in two-way repeated-measures ANOVAs with tDCS condition (3) and block (3) as within-subjects factors. Novel faces RTs and accuracy were entered in one-way repeated-measures ANOVAs with tDCS condition (3) as within-subjects factor (no block factor can be assigned to novel faces).

ERP analyses were conducted for latency and amplitude values of the P100, N170 and P3a components. Peaks were individually detected for all trial averages (Av1, Av2 and Av3) in a ±30ms time window around the grand-average means at Oz, O1 and O2 sites for P100, TP8 and TP7 for N170 and along the midline (Fz, FCz, Cz, CPz, Pz) for P3a. A four-way repeated-measures ANOVA was conducted on P100 data, using electrode (3), tDCS condition (3), block (3) and trial average (3) as within-subjects factors. For the N170 component, a four-way repeated-measures ANOVA was carried out with hemisphere (2), stimulation condition (3), block (3) and trial average (3) as within-subjects factors. The P3a component was analyzed using a four-way, repeated-measures ANOVA with electrode (5), stimulation condition (3), block (3) and trial average (3) as within-subjects factors. When obtained, main effects were further analyzed at specific factor levels. Interaction effects were analyzed with interaction-graph guided ANOVAs at specific levels of the independent variables. All statistical analyses involved Greenhouse-Geisser adjusted degrees of freedom when necessary and Bonferroni corrections for multiple comparisons.

## Results

### Behavioural results

**PSQI.** Descriptive statistics indicate a mean global score of 3.86±0.29 (range: 2–5) which is within recommended "good" sleeping quality values (≤5) [70]. This suggests none of the participants had significant sleeping disturbances for the entire duration of testing (10 days, all conditions) and within the preceding month.

**tDCS questionnaire.** Participants reported some adverse side-effects due to tDCS. During stimulation, 93% of participants reported an "itching" sensation under the electrodes and 78.5% reported "burning". Such side-effects were anticipated as they are relatively common in tDCS studies [63]. Furthermore, on average, these sensations were rated as "mild" and in no case entailed lesions or motivated participants to withdraw from further testing.

**Recognition task accuracy.** Recognition task difficulty was set to yield accuracies lower than ceiling to allow for improvement and decrement of performance. The range of accuracy rates was 51-80% (mean ± SD: 61%±7%), all conditions, participants and types of stimuli (encoded vs. novel) included. No effect of tDCS condition (*F*(2,20) = 1.13, *P*>0.1, η_p_^2^ = 0.1), stimulus familiarity (*F*(1,10) = 2.23, *P*>0.1, η_p_^2^ = 0.18) or interaction (*F*(2,22) = 0.76, *P*>0.1, η_p_^2^ = 0.07) were found on recognition accuracy.

**Figure 2:**
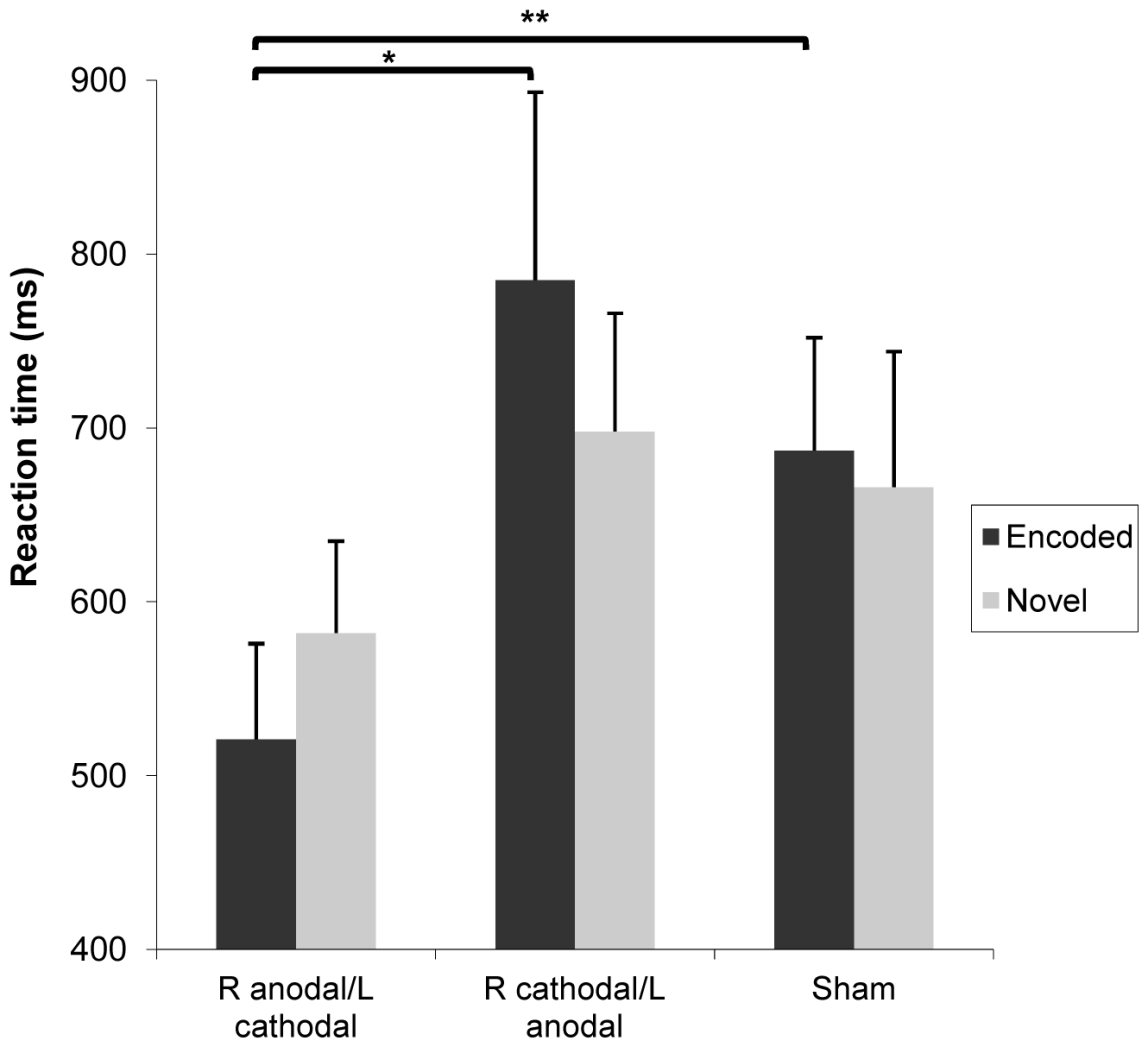
Effect of tDCS condition on recognition RTs. For faces encoded while under tDCS influence, the right anodal/left cathodal condition yielded faster recognition RTs than the right cathodal/left anodal (*P<0.05) and sham conditions (**P<0.01), ∼72 hours after encoding. There was no effect of tDCS on RTs of novel faces.

**Recognition task RTs.** A marginally significant main effect of tDCS condition was found on overall RTs at recognition (*F*(2,20) = 3.2, *P* = 0.06, η_p_^2^ = 0.24), but not of stimulus familiarity (*F*(1,10) = 1.5, *P*>0.1, η_p_^2^ = 0.13) or interaction (*F*(2,20) = 0.64, *P*>0.1, η_p_^2^ = 0.06). For encoded stimuli, a main effect of tDCS condition was found (*F*(1.23,12.26) = 4.2, *P*<0.05, η_p_^2^ = 0.29), but not of block (*F*(1.19,11.87) = 1.54, *P*>0.1, η_p_^2^ = 0.13) or interaction (*F*(1.13,11.33) = 0.6, *P*>0.1, η_p_^2^ = 0.06). Indeed, Bonferroni-adjusted pairwise comparisons indicate RTs following the right anodal/left cathodal condition were faster than both the right cathodal/left anodal (*P<*0.05) and sham conditions (*P<*0.01). For novel faces of the recognition task, no tDCS condition effect was found on RTs (*F*(2,20) = 2.2, *P*>0.1, η_p_^2^ = 0.18), indicating that the effect of tDCS condition on RTs is confined to stimuli presented following active tDCS (Figure 2). Single tDCS sessions of the DLPFC during encoding were sufficient to induce long-term differential effects on recognition RTs as a function of polarity. Moreover, as there was no encoding block effect, the tDCS-induced effects on RTs seem to have lasted the entire duration of the encoding task over all three blocks.

### ERP results

**P100.** All interactions were non-significant (*P>*0.1) and no main effect of electrode (*F*(2,20) = 1.63, *P*>0.1, η_p_^2^ = 0.14), stimulation condition (*F*(2,20) = 2.3, *P*>0.1, η_p_^2^ = 0.2), block (*F*(2,20) = 0.4, *P*>0.1, η_p_^2^ = 0.04) or trial average (*F*(2,20) = 0.3, *P*>0.1, η_p_^2^ = 0.01) was found for amplitude values of the P100 at posterior sites. For latency values, no electrode (*F*(2,20) = 1.1, *P*>0.1, η_p_^2^ = 0.1), tDCS condition (*F*(2,20) = 0.23, *P*>0.1, η_p_^2^ = 0.02), block (*F*(2,20) = 2.15, *P*>0.1, η_p_^2^ = 0.2), trial average (*F*(2,20) = 0.46, *P*>0.1, η_p_^2^ = 0.02) effects or interactions (*P>*0.1) were significant.

**Figure 3:**
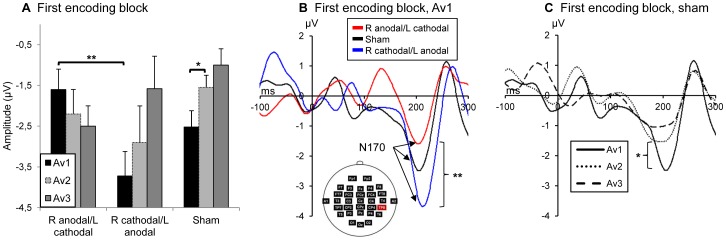
Effects of tDCS condition and trial average on N170 amplitude over right occipito-temporal cortex. (A) Graph showing a tDCS condition x trial average interaction on N170 amplitude within the first encoding block. (B) This interaction further reveals an effect of tDCS condition on N170 amplitude within the first five stimulus repetitions (Av1) of the first encoding bloc, where the R anodal/L cathodal condition is significantly suppressed relative to the R cathodal/L anodal condition (**P = 0.01). (C) A trial average effect in the sham condition reveals RS of the N170 from Av1 to Av2 (*P = 0.05). No trial average effects were found for both the right anodal/left cathodal and right cathodal/left anodal conditions.

**N170.** For amplitude values, an interaction effect of hemisphere x tDCS condition x block x trial average was found (*F*(8,80) = 2.12, *P*<0.05, η_p_^2^ = 0.18). When hemispheres were analyzed separately, no main or interaction effects were found at left hemisphere electrode TP7 (*P>*0.1) however, right occipito-temporal site TP8 (Table 1) showed a significant tDCS condition x block x trial average interaction effect (*F*(8,80) = 2.62, *P* = 0.01, η_p_^2^ = 0.21). In turn, analyzing encoding blocks separately at TP8, revealed a significant tDCS condition x trial average effect (*F*(4,40) = 4.73, *P*<0.01, η_p_^2^ = 0.32) specific to the first encoding block (Figure 3A). A significant tDCS condition effect (*F*(2,20) = 11.2, *P* = 0.01, η_p_^2^ = 0.53) was found to be circumscribed to the first five trials (Av1) within the first encoding block at TP8. Indeed, pairwise comparisons indicate that N170 amplitude is significantly suppressed for the right anodal/left cathodal condition relative to the right cathodal/left anodal condition (*P = *0.01, Figure 3A,B). Similarly to RT results, sham condition N170 amplitude lied in between those of active tDCS conditions, these differences being marginally significant (*P<*0.08). All other blocks and trial averages showed non-significant tDCS condition effects (*P*>0.1).

**Table 1:** Grand-averaged N170 amplitudes (mV) at right occipito-temporal site TP8.

	Block 1			Block 2			Block 3		
	Av1	Av2	Av3	Av1	Av2	Av3	Av1	Av2	Av3
**R anodal/L cathodal**	–1.6 (0.5)	–2.2 (0.6)	–2.5 (0.5)	–2.2 (1.2)	–0.98 (0.92)	–0.16 (0.97)	–2.23 (0.79)	–2.53 (1.04)	–1.84 (1.1)
**R cathodal/L anodal**	–3.72 (0.6)	–2.9 (0.9)	–1.58 (0.8)	–1.97 (1.12)	–3.44 (1.01)	–2.18 (0.48)	–1.58 (0.94)	–3.68 (1.24)	–3.32 (1.02)
**Sham**	–2.52 (0.4)	–1.55 (0.3)	–1.03 (0.4)	–3.08 (0.98)	–1.25 (0.8)	–1.44 (0.84)	–1.27 (0.95)	–2.87 (1.14)	–2.39 (1.06)

Data are presented as mean and SEM.

Of special interest here, the tDCS condition effect on N170 amplitude at Av1 of the first encoding block suggests that differential right occipito-temporal RS took place between active stimulation conditions within the first five stimulus repetitions of the first block, when active tDCS modulatory influence on DLPFC excitability was presumably strongest. This supports the hypothesis that tDCS over the DLPFC would modulate N170 RS over occipito-temporal sites in response to repeated presentation of unfamiliar faces. However, as this is only inferred from observing an overall difference between resulting averages (i.e.: Av1), additional analyses were conducted to ensure the tDCS condition effect can be interpreted in the context of RS. First, to ascertain that the encoding task effectively induced RS in the absence of DLPFC activity modulation, the amplitude values of N170 should decrease as a function of trial average within the first block of the sham condition. In an analysis with tDCS condition (3) and trial average (3) as within-subjects factors, a significant interaction was found (*F*(4,40) = 4.73, *P*<0.01, η_p_^2^ = 0.32). As expected, a trial average effect was revealed (*F*(2,20) = 3.46, *P<*0.05, η_p_^2^ = 0.26) in the sham condition where N170 amplitude reduced from Av1 to Av2 (*P = *0.05, Figure 3A,C). Although Av3 N170 amplitude remains reduced relative to Av1, this difference was not significant (*P>*0.1). Trial average effects were non-significant in the right anodal/left cathodal (*F*(2,20) = 2.62, *P* = 0.1, η_p_^2^ = 0.21) and right cathodal/left anodal (*F*(2,20) = 2.37, *P*>0.1, η_p_^2^ = 0.19) conditions. Secondly, to confirm that the tDCS effect suppresses activity rather than creating a non specific change in brain activity, the amplitude values of the very first presentations of each face of the first encoding block would have to be equal across conditions. This was in fact the case as no significant difference was found in a supplementary one-way repeated-measures ANOVA with tDCS condition as within-subjects factor and grand-averaged N170 amplitude values of the first stimulus presentation as outcome variable (*F*(2,24) = 0.32, *P*>0.1, η_p_^2^ = 0.03). It follows from this analysis, that the tDCS condition effect at Av1 of the first block would be caused by a difference in the last four repetitions of Av1. Indeed, the tDCS condition effect remains when grand-averaged amplitude values of only the last four repetitions of Av1 are included (*F*(2,20) = 7.49, *P*<0.01, η_p_^2^ = 0.43). Together, these results demonstrate that the tDCS condition effect on N170 amplitude reflects differential RS between the active tDCS conditions during the first five repetitions of stimuli. Furthermore, the fact that the tDCS condition effect was specific to the right hemisphere where face stimuli are preferentially processed [73], [74], suggests that this effect was specifically induced by repeated presentation of unfamiliar faces.

For latency values of the N170 component, all interactions were non-significant (*P*>0.1). No main effects of hemisphere (*F*(1,10) = 1.9, *P>*0.1, η_p_^2^ = 0.16), stimulation condition (*F*(2,20) = 2.67, *P = *0.1, η_p_^2^ = 0.21), block (*F*(1,10) = 1.9, *P>*0.1, η_p_^2^ = 0.16) or trial average (*F*(2,20) = 1.1, *P>*0.1, η_p_^2^ = 0.1) were found.

**Figure 4:**
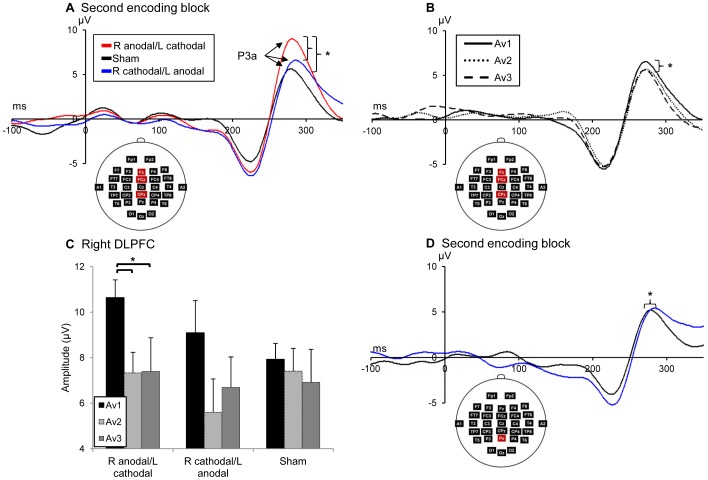
Effects of tDCS condition on grand averaged P3a amplitudes and latencies. (A) The right anodal/left cathodal condition caused larger P3a amplitudes than the right cathodal/left anodal. This effect was strongest at the second encoding block (displayed, *P<0.05). (B) P3a amplitude suppressed from Av1 to Av3 regardless of tDCS condition and block, indicating RS throughout repetitions of stimuli (*P<0.05). (C) Effect of tDCS condition x trial average interaction at right dorsolateral site F4 where RS is significant only in the right anodal/left cathodal condition (*P<0.05). (D) The right cathodal/left anodal condition caused a significant delay of P3a at Pz (*P<0.05).

**P3a.** While all interactions were non-significant (*P*>0.1), main effects of stimulation condition (*F*(2,20) = 3.85, *P<*0.05, η_p_^2^ = 0.29) and trial average (*F*(2,9) = 4.87, *P<*0.05, η_p_^2^ = 0.52) were found on amplitude values of the P3a component. The tDCS condition effect was caused by overall higher P3a amplitude in the right anodal/left cathodal condition relative to the inversed polarity and sham conditions (*P<*0.05, Figure 4A). Also, Av1 amplitudes were higher than Av3 regardless of stimulation condition and block (*P*<0.05), indicating suppression of activity throughout repetitions (1–15) of single stimuli (Figure 4B). When sites were analyzed separately, similar tDCS condition effects were found individually at Fz (*F*(2,20) = 4.33, *P<*0.05, η_p_^2^ = 0.3), FCz (*F*(2,20) = 4.41, *P<*0.05, η_p_^2^ = 0.31) and CPz (*F*(2,9) = 4.38, *P<*0.05, η_p_^2^ = 0.49). These stimulation condition effects were driven by the second block at these sites (Fz: *F*(1.3,13.1) = 6.99, *P<*0.01, η_p_^2^ = 0.41; FCz: *F*(2,20) = 7.66, *P<*0.01, η_p_^2^ = 0.43; CPz: *F*(2,20) = 5.83, *P = *0.01, η_p_^2^ = 0.37). Indeed, the first and third blocks showed no tDCS condition effect (*P*>0.05). Trial average or RS effects also remained significant at Fz (*F*(2,9) = 5.58, *P<*0.05, η_p_^2^ = 0.55), FCz (*F*(2,9) = 6.88, *P<*0.05, η_p_^2^ = 0.6) and CPz (*F*(2,9) = 4.81, *P<*0.05, η_p_^2^ = 0.52).

As they were the central sites over which tDCS electrodes were placed, frontal dorsolateral sites F4 and F3 were analyzed in a separate four-way repeated-measures ANOVA with hemisphere (2), tDCS condition (3), block (3) and trial average (3) as within-subjects factors. In this analysis, a significant main effect of trial average was found (*F*(2,9) = 6.33, *P<*0.05, η_p_^2^ = 0.58). This trial average effect was significant at both F3 (*F*(2,9) = 4.54, *P<*0.05, η_p_^2^ = 0.5) and F4 (*F*(2,9) = 5.96, *P<*0.05, η_p_^2^ = 0.57), where an additional interaction of tDCS condition x trial average was found (*F*(4,7) = 1.91, *P<*0.05, η_p_^2^ = 0.52). This interaction further revealed that a significant RS effect was present only in the right anodal/left cathodal condition at this site (*F*(2,20) = 4.1, *P*<0.05, η_p_^2^ = 0.3) between Av1 and Av2, as well as Av1 and Av3 (*P*<0.05, Figure 4C). These results suggest that while having the same neurophysiological influence (i.e.: cortical excitability augmentation for the anode), tDCS polarity did not have the same induced effects on RS of the P3a depending on the hemisphere over which it was applied.

Latency values analyses revealed a general trend for P3a to be delayed in the right cathodal/left anodal condition at all midline and frontal dorsolateral sites. However, this main effect was significant only at Pz (*F*(2,20) = 3.76, *P*<0.05, η_p_^2^ = 0.27), specifically between the right cathodal/left anodal and sham conditions at the second block (*P*<0.05, Figure 4D).

## Discussion

The purpose of the present study was to determine whether prefrontal regions, the DLPFC specifically, can, through top-down control, influence early visual encoding of individual face identities triggered by repeated viewing of unfamiliar faces. To do so, we exogenously modulated DLPFC activity using tDCS in healthy adults before encoding of unfamiliar faces, and analyzed RS variations in the P100, N170 and P3a ERP components, as well as long-term recognition accuracy and RT. Immediately following tDCS, ERP data revealed differential effects in N170 amplitude values for averaged repeated stimuli between conditions where prefrontal cortical activity was oppositely altered (i.e.: polarity-dependent). This suggests that neural activity to repeated face identity processing in right occipito-temporal cortex was modulated through prefrontal activity disruption and enhancement. tDCS did modulate prefrontal activity, as concurrent effects on both amplitude and latency values of the P3a component reflected variations in prefrontal function. Notably, P3a RS interacted with tDCS condition over the right hemisphere, further supporting the DLPFC’s influence on RS. DLPFC stimulation also influenced behaviour, as RTs in subsequent recognition tasks followed a polarity-dependent pattern in line with ERP results. These data support the hypothesis that top-down connections may causally influence early visual encoding and extend it by showing a specific link between DLPFC activity modulation by tDCS and right-hemisphere N170 RS, having long-term repercussions on recognition.

Our main behavioural finding was that participants made faster decisions about the familiarity of face stimuli encoded under the right anodal/left cathodal condition than under the right cathodal/left anodal. This effect held for all three encoding blocs and only for encoded faces, which enforces the notion that this variation is indeed tDCS-induced. There is strong evidence from studies on priming that leads us to expect facilitation of processing such as faster RT for previously encoded stimuli [1], [5], [6], [7]. Thus, the observation that RTs are shortened in the right anodal/left cathodal condition relative to sham suggests that facilitation of processing is enhanced in this condition. Reversal of tDCS polarity had the opposite effect on RTs which were longer than in all other conditions but only significantly so relative to the right anodal/left cathodal. This suggests that the right cathodal/left anodal condition negatively impacted expected priming or at least did not confer the same advantage as did the right anodal/left cathodal condition. No such advantage or impairment was obtained in recognition accuracy, as scores were equivalent across conditions. At encoding, latency of presentation (2000ms) or the number of presentations (15 for each individual identity) may have been long enough or sufficient to override effects on accuracy that could have been induced by tDCS. Similar findings have been reported by Itier and Taylor [17] where encoding was facilitated enough to abolish the expected higher retrieval accuracy for upright relative to inverted and negative-contrast faces.

In the absence of tDCS influence, the N170 showed RS, as Av2 trial amplitude was significantly reduced relative to Av1. Moreover, although amplitude was equal across conditions for the first presentation of individual face identities, N170 average amplitude of the first five repetitions (Av1) varied as a function of tDCS polarity, indicating that different activity patterns took place within these first repetitions. Indeed, although our analyses do not provide a trial-by-trial resolution, it seems the right anodal/left cathodal condition induced a progressive suppression of activity in response to repeated face stimuli, whereas the right cathodal/left anodal condition increased activity, as reflected by its larger amplitude. Additionally, effects on N170 RS were significant at the first encoding block and fell thereafter, which suggests tDCS was responsible for early face identity processing effects over occipito-temporal cortex: as DLPFC activity modulation due to electrical current presumably faded, so did N170 RS modulation. This is comparable to results obtained by Zanto and colleagues [31], whose effects of prefrontal disruption by TMS on posterior areas and working memory waned as time elapsed. The gradual reduction of N170 amplitude under the right anodal/left cathodal condition is congruous with faster RTs in this condition, given previous studies that have found RS and N170 RS, to be associated with positive priming and familiarity acquisition effects for unfamiliar faces [2], [22], [25], [39]. Conversely, enlargement of N170 amplitude has typically been linked with higher processing and attentional demands due to an increase of task difficulty. A well established example of N170 amplitude increase is in the response to presentation of inverted faces which disrupt normal ease of configural processing for upright faces [18], [19], [75]. This in turn requires that existing neural mechanisms deploy greater efforts or that additional mechanisms contribute to processing, resulting in higher signal amplitude and severely impaired learning capacity [76]. Enlargement of N170 amplitude has also been reported to occur as a function of increased working memory load for faces. In a task where the number of faces having to be simultaneously encoded is incrementally increased, so is N170 amplitude, again thought to reflect a higher demand of neural resources [77]. Following Friston’s theory [39], an increase of activity could reflect an inability of higher cortical areas to reduce prediction error with repetition. This may explain why N170 amplitude can increase when the second face of a pair is of a different identity than the first [22]: identity processing must begin anew and prediction error is increased relative to the first identity. Similarly, perhaps N170 amplitude does not reduce for familiar faces [24] because once an identity is learned, RS and prediction error reach a minimum. In the present study, despite the fact that only one face was presented at a time, that stimuli were upright, unfamiliar and of the same identity within Av1, N170 amplitude still increased in the right cathodal/left anodal condition. Moreover, accuracy being equal, more time was needed to recognize these faces afterwards. In light of past findings and our behavioural results, N170 amplitude enhancement in this study may indicate compromised efficacy of visual face identity encoding elicited by the right cathodal/left anodal condition. The inverse may be said of the right anodal/left cathodal condition.

Heightened P3a amplitude, found in the right anodal/left cathodal condition, can reflect high availability and focus of attentional resources, which has generally been linked to enhanced encoding and subsequent retrieval ability [78], [79], [44]. Oppositely, when a task is more demanding of attentional resources, such as one requiring high working memory load for faces, general P300 amplitudes tend to be reduced as attentional resources become depleted [77], [43]. Furthermore, latency delay of the P3a found in the right cathodal/left anodal condition, has been associated with increased demand of attentional resources and cognitive processing in difficult tasks [80], [43]. In this study, task difficulty and working memory load did not vary across conditions. Consequently, the right cathodal/left anodal condition may have effectively compromised neuronal and attentional resources, thereby artificially increasing the encoding task’s difficulty. Thus, the influence of tDCS on P3a amplitude seems to be in line with the notion of encoding efficacy decrease for the right cathodal/left anodal condition and increase for the right anodal/left cathodal.

What remains uncertain is the exact mechanism through which tDCS has induced effects in this study. Improvement of many aspects of learning have been demonstrated using anodal stimulation [59], [81], [82], which seems to provoke long-term potentiation (LTP)-like mechanisms by modulating NMDA receptor plasticity [50]–[52]. Hence, anodal stimulation may have increased synaptic potentiation of existing connections between DLPFC and occipito-temporal cortex, leading to progressively more efficient and faster interaction between these regions, which is in agreement with a "facilitation" model of RS [4], [39]. In contrast, cathodal stimulation reduces cortical excitability and usually has a negative or no impact on learning [52], [58], [59]. Reconciling these findings with the present results leads us to a prevalent importance of the right DLPFC in influencing face identity encoding. Regardless of left DLPFC stimulation, positive priming and RS effects on N170 were specific to the right occipito-temporal cortex when the anode was above the right DLPFC and the opposite for the cathode. Moreover, the P3a’s RS effect interacted with tDCS polarity over the right DLPFC while no such interaction was present over the left DLPFC. This may be the consequence of a combination of right hemisphere preference for faces [73], [74] and intrahemispheric prefrontal modulation of occipito-temporal cortex [28]. However, through the necessity of having simultaneous presence of anodal and cathodal stimulation over areas of cortex, tDCS effects pose an inherent difficulty of interpretation in determining which stimulation causes the effects, if not a combination of the two, as proposed by Fecteau et al. [55]. Additionally, given that N170 and P3a effects were maximal at the first and second blocks respectively, while RT effects were found in all three encoding blocks, the mechanisms underlying ERPs investigated here at encoding may not be sufficient in accounting for influences on behaviour.

Although N170 peak latencies of up to 240ms have been confidently reported (e.g.: [83]), the ones obtained in this study (∼210ms) are somewhat longer than the typical time-range (130–200ms) [84]. Certain factors can explain this peculiarity. The fact that the face stimuli used here were cropped (showing only the inside features of faces) may have increased N170 peak latency, as it did in Dering and colleagues’ study [85], when compared to unaltered faces. Longer N170 latency may also be a result of the "SHINEing" of stimuli which strongly reduced variations in low-level image properties including spatial frequency. Some studies have found large delays of N170 latency in response to spatial frequency filtered face stimuli [86], [87]. However, this is the first study to report N170 effects from stimuli having undergone the SHINE process and further investigation is needed to determine its specific impacts. Finally, repetition itself may have also contributed to N170 latencies. While some studies have found N170 amplitude reduction to be associated with shorter latencies [18], [19], others have found either no latency variations [15], [20] or increased latency with repetition [88]. N170 latency patterns in response to repetition could be more vulnerable to task design factors (e.g.: presentation duration, intertrial interval, presence and number of intervening items) which are highly variable across studies on RS. Further study is needed to gain insight into this matter.

In conclusion, results presented here demonstrate that tDCS of the DLPFC can influence early visual encoding of individual face identities indexed by N170 RS. The fact that reversal of tDCS polarity over the DLPFC yielded differences in prefrontal function, N170 repetition effects and long-term recognition RT, suggests that visual encoding efficacy has the potential to be either enhanced or impaired through DLPFC stimulation.

## References

[1] Henson RNA (2003) Neuroimaging studies of priming. Prog Neurobiol 70: 53–81. 12927334 10.1016/s0301-0082(03)00086-8

[2] Sayres R, Grill-Spector K (2006) Object-selective cortex exhibits performance-independent repetition suppression. J Neurophysiol 95: 995–1007. 16236787 10.1152/jn.00500.2005

[3] Desimone R (1996) Neural mechanisms for visual memory and their role in attention. Proc Natl Acad Sci U S A 93: 13494–99. 8942962 10.1073/pnas.93.24.13494 PMC33636

[4] Grill-Spector K, Henson R, Martin A (2006) Repetition and the brain: neural models of stimulus-specific effects. Trends Cogn Sci 10: 14–23. 16321563 10.1016/j.tics.2005.11.006

[5] Schacter DL, Buckner RL (1998) Priming and the brain. Neuron 20: 185–195. 9491981 10.1016/s0896-6273(00)80448-1

[6] Tulving E, Schacter DL (1990) Priming and human memory systems. Science 247: 301–306. 2296719 10.1126/science.2296719

[7] Wiggs CL, Martin A (1998) Properties and mechanisms of perceptual priming. Curr Opin Neurobiol 8: 227–233. 9635206 10.1016/s0959-4388(98)80144-x

[8] Zago L, Fenske MJ, Aminoff E, Bar M (2005) The rise and fall of priming: how visual exposure shapes cortical representations of objects. Cereb Cortex 15: 1655–1665. 15716471 10.1093/cercor/bhi060 PMC1564465

[9] Gonsalves BD, Kahn I, Curran T, Norman KA, Wagner AD (2005) Memory strength and repetition suppression: multimodal imaging of medial temporal cortical contributions to recognition. Neuron 47: 751–761. 16129403 10.1016/j.neuron.2005.07.013

[10] Pihlajamaki M, O'Keefe K, O'Brien J, Blacker D, Sperling RA (2011) Failure of repetition suppression and memory encoding in aging and Alzheimer's disease. Brain Imaging Behav 5: 36–44. 21161449 10.1007/s11682-010-9110-3 PMC3034137

[11] Schiltz C, Sorger B, Caldara R, Ahmed F, Mayer E, et al (2006) Impaired face discrimination in acquired prosopagnosia is associated with abnormal response to individual faces in the right middle fusiform gyrus. Cereb Cortex 16: 574–586. 16033923 10.1093/cercor/bhj005

[12] Williams MA, Berberovic N, Mattingley JB (2007) Abnormal FMRI adaptation to unfamiliar faces in a case of developmental prosopamnesia. Curr Biol 17: 1259–1264. 17614283 10.1016/j.cub.2007.06.042

[13] Bentin S, Allison T, Puce A, Perez E, McCarthy G (1996) Electrophysiological studies of face perception in humans. J Cogn Neurosci 8: 551–565. 20740065 10.1162/jocn.1996.8.6.551 PMC2927138

[14] Rossion B, Gauthier I, Tarr MJ, Despland P, Bruyer R, et al (2000) The N170 occipito-temporal component is delayed and enhanced to inverted faces but not inverted objects: An electrophysiological account of face-specific processes in the human brain. Neuroreport 11: 69–72. 10683832 10.1097/00001756-200001170-00014

[15] Heisz JJ, Watter S, Shedden JM (2006) Progressive N170 habituation to unattended repeated faces. Vision Res 46: 47–56. 16289274 10.1016/j.visres.2005.09.028

[16] Campanella S, Hanoteau C, Dépy D, Rossion B, Bruyer R, et al (2000) Right N170 modulation in a face discrimination task: an account for categorical perception of familiar faces. Psychophysiology 37: 796–806. 11117460

[17] Rotshtein P, Henson RN, Treves A, Driver J, Dolan RJ (2005) Morphing Marilyn into Maggie dissociates physical and identity face representations in the brain. Nat Neurosci 8: 107–113. 15592463 10.1038/nn1370

[18] Itier RJ, Taylor MJ (2002) Inversion and contrast polarity reversal affect both encoding and recognition processes of unfamiliar faces: a repetition study using ERPs. Neuroimage 15: 353–372. 11798271 10.1006/nimg.2001.0982

[19] Itier RJ, Taylor MJ (2004) Effects of repetition learning on upright, inverted and contrast-reversed face processing using ERPs. Neuroimage 21: 1518–1532. 15050576 10.1016/j.neuroimage.2003.12.016

[20] Heisz JJ, Watter S, Shedden JM (2006) Automatic face identity encoding at the N170. Vision Res 46: 4604–4614. 17097126 10.1016/j.visres.2006.09.026

[21] Maurer D, Le Grand R, Mondloch CJ (2002) The many faces of configural processing. Trends Cogn Sci 6: 255–260. 12039607 10.1016/s1364-6613(02)01903-4

[22] Caharel S, d'Arripe O, Ramon M, Jacques C, Rossion B (2009) Early adaptation to repeated unfamiliar faces across viewpoint changes in the right hemisphere: evidence from the N170 ERP component. Neuropsychologia 47: 639–643. 19084547 10.1016/j.neuropsychologia.2008.11.016

[23] Caharel S, Jacques C, d'Arripe O, Ramon M, Rossion B (2011) Early electrophysiological correlates of adaptation to personally familiar and unfamiliar faces across viewpoint changes. Brain Res 1387: 85–98. 21362409 10.1016/j.brainres.2011.02.070

[24] Heisz JJ, Shedden JM (2008) Semantic learning modifies perceptual face processing. J Cogn Neurosci 21: 1127–1134. 10.1162/jocn.2009.21104 18752406

[25] Vizioli L, Rousselet GA, Caldara R (2010) Neural repetition suppression to identity is abolished by other-race faces. Proc Natl Acad Sci U S A 107: 20081–20086. 21041643 10.1073/pnas.1005751107 PMC2993371

[26] Hillyard SA, Anllo-Vento L (1998) Envent-related brain potentials in the study of visual selective attention. Proc Natl Acad Sci U S A 95: 781–787. 9448241 10.1073/pnas.95.3.781 PMC33798

[27] Miller EK, Cohen JD (2001) An integrative theory of prefrontal cortex function. Annu Rev Neurosci 24: 167–202. 11283309 10.1146/annurev.neuro.24.1.167

[28] Barcelò F, Suwazono S, Knight RT (2000) Prefrontal modulation of visual processing in humans. Nat Neurosci 3: 399–403. 10725931 10.1038/73975

[29] Chadick JZ, Gazzaley A (2011) Differential coupling of visual cortex with default or frontal-parietal network based on goals. Nat Neurosci 14: 830–832. 21623362 10.1038/nn.2823 PMC3125492

[30] Kuo BC, Yeh YY, Chen AJ, D'Esposito M (2011) Functional connectivity during top-down modulation of visual short-term memory representations. Neuropsychologia 49: 1589–1596. 21241721 10.1016/j.neuropsychologia.2010.12.043 PMC3085092

[31] Zanto TP, Rubens MT, Thangavel A, Gazzaley A (2011) Causal role of the prefrontal cortex in top-down modulation of visual processing and working memory. Nat Neurosci 14: 656–661. 21441920 10.1038/nn.2773 PMC3083493

[32] Habib R, Nyberg L, Tulving E (2003) Hemispheric asymmetries of memory: the HERA model revisited. Trends Cogn Sci 7: 241–245. 12804689 10.1016/s1364-6613(03)00110-4

[33] Tulving E, Kapur S, Craik FIM, Moscovitch M, Houle S (1994) Hemispheric encoding/retrieval asymmetry in episodic memory: Positron emission tomography findings. Proc Natl Acad Sci U S A 91: 2016–2020. 8134342 10.1073/pnas.91.6.2016 PMC43300

[34] Hofer A, Siedentopf CM, Ischebeck A, Rettenbacher MA, Verius M, et al (2007) Neural substrates for episodic encoding and recognition of unfamiliar faces. Brain Cogn 63: 174–181. 17207899 10.1016/j.bandc.2006.11.005

[35] Sergerie K, Lepage M, Armony JL (2005) A face to remember: emotional expression modulates prefrontal activity during memory formation. Neuroimage 24: 580–585. 15627601 10.1016/j.neuroimage.2004.08.051

[36] Ishai A (2008) Let's face it: it's a cortical network. Neuroimage 40: 415–419. 18063389 10.1016/j.neuroimage.2007.10.040

[37] Büchel C, Coull JT, Friston KJ (1999) The Predictive Value of Changes in Effective Connectivity for Human Learning. Science 283: 1538–1541. 10066177 10.1126/science.283.5407.1538

[38] Dale AM, Liu AK, Fischl BR, Buckner RL, Belliveau JW, et al (2000) Dynamic statistical parametric mapping: Combining fMRI and MEG for high-resolution imaging of cortical activity. Neuron 26: 55–67. 10798392 10.1016/s0896-6273(00)81138-1

[39] Friston K (2005) A theory of cortical responses. Philos Trans R Soc Lond B Biol Sci 360: 815–836. 15937014 10.1098/rstb.2005.1622 PMC1569488

[40] Summerfield C, Trittschuh EH, Monti JM, Mesulam MM, Egner T (2008) Neural repetition suppression reflects fulfilled perceptual expectations. Nat Neurosci 11: 1004–1006. 19160497 10.1038/nn.2163 PMC2747248

[41] Kovacs G, Iffland L, Vidnyanszky Z, Greenlee MW (2012) Stimulus repetition probability effects on repetition suppression are position invariant for faces. Neuroimage 60: 2128–2135. 22387172 10.1016/j.neuroimage.2012.02.038

[42] Kovacs G, Kaiser D, Kaliukhovich DA, Vidnyanszky Z, Vogels R (2013) Repetition Probability Does Not Affect fMRI Repetition Suppression for Objects. J Neurosci 33: 9805–9812. 23739977 10.1523/JNEUROSCI.3423-12.2013 PMC6619695

[43] Polich J (2007) Updating P300: an integrative theory of P3a and P3b. Clin Neurophysiol 118: 2128–2148. 17573239 10.1016/j.clinph.2007.04.019 PMC2715154

[44] Ranganath C, Rainer G (2003) Neural mechanisms for detecting and remembering novel events. Nat Rev Neurosci 4: 193–202. 12612632 10.1038/nrn1052

[45] Daffner KR, Mesulam MM, Scinto LFM, Acar D, Calvo V, et al.. (2000) The central role of the prefrontal cortex in directing attention to novel events. Brain 927–939. 10.1093/brain/123.5.927 10775538

[46] Woods DL, Knight RT (1986) Electrophysiologic evidence of increased distractibility after dorsolateral prefrontal lesions. Neurology 36: 212–216. 3945393 10.1212/wnl.36.2.212

[47] Friedman D, Cycowicz YM, Gaeta H (2001) The novelty P3: an event-related brain potential (ERP) sign of the brain's evaluation of novelty. Neurosci Biobehav Rev 25: 355–373. 11445140 10.1016/s0149-7634(01)00019-7

[48] Friedman D, Nessler D, Kulik J, Hamberger M (2011) The brain's orienting response (novelty P3) in patients with unilateral temporal lobe resections. Neuropsychologia 49: 3474–3483. 21906606 10.1016/j.neuropsychologia.2011.08.023 PMC3211086

[49] Nitsche MA, Paulus W (2000) Excitability changes induced in the human motor cortex by weak transcranial direct current stimulation. J Physiol 15: 633–639. 10.1111/j.1469-7793.2000.t01-1-00633.x PMC2270099 10990547

[50] Nitsche MA, Seeber A, Frommann K, Klein CC, Rochford C, et al (2005) Modulating parameters of excitability during and after transcranial direct current stimulation of the human motor cortex. J Physiol 568: 291–303. 16002441 10.1113/jphysiol.2005.092429 PMC1474757

[51] Sparing R, Mottaghy FM (2008) Noninvasive brain stimulation with transcranial magnetic or direct current stimulation (TMS/tDCS)-From insights into human memory to therapy of its dysfunction. Methods 44: 329–337. 18374276 10.1016/j.ymeth.2007.02.001

[52] Wassermann EM, Grafman J (2005) Recharging cognition with DC brain polarization. Trends Cogn Sci 9: 503–505. 16182596 10.1016/j.tics.2005.09.001

[53] Dockery CA, Hueckel-Weng R, Birbaumer N, Plewnia C (2009) Enhancement of planning ability by transcranial direct current stimulation. J Neurosci 29: 7271–7277. 19494149 10.1523/JNEUROSCI.0065-09.2009 PMC6666475

[54] Boggio PS, Campanha C, Valasek CA, Fecteau S, Pascual-Leone A, et al (2010) Modulation of decision-making in a gambling task in older adults with transcranial direct current stimulation. Eur J Neurosci 31: 593–597. 20105234 10.1111/j.1460-9568.2010.07080.x

[55] Fecteau S, Pascual-Leone A, Zald DH, Liguori P, Theoret H, et al (2007) Activation of prefrontal cortex by transcranial direct current stimulation reduces appetite for risk during ambiguous decision making. J Neurosci 27: 6212–6218. 17553993 10.1523/JNEUROSCI.0314-07.2007 PMC6672163

[56] Fecteau S, Knoch D, Fregni F, Sultani N, Boggio P, et al (2007) Diminishing risk-taking behavior by modulating activity in the prefrontal cortex: a direct current stimulation study. J Neurosci 27: 12500–12505. 18003828 10.1523/JNEUROSCI.3283-07.2007 PMC6673338

[57] Mulquiney PG, Hoy KE, Daskalakis ZJ, Fitzgerald PB (2011) Improving working memory: exploring the effect of transcranial random noise stimulation and transcranial direct current stimulation on the dorsolateral prefrontal cortex. Clin Neurophysiol 122: 2384–2389. 21665534 10.1016/j.clinph.2011.05.009

[58] Elmer S, Burkard M, Renz B, Meyer M, Jancke L (2009) Direct current induced short-term modulation of the left dorsolateral prefrontal cortex while learning auditory presented nouns. Behav Brain Funct 5: 29. 19604352 10.1186/1744-9081-5-29 PMC2719658

[59] Flöel A, Rösser N, Michka O, Knecht S, Breitenstein C (2008) Noninvasive brain stimulation improves language learning. J Cogn Neurosci 20: 1415–1422. 18303984 10.1162/jocn.2008.20098

[60] Cohen Kadosh R, Soskic S, Iuculano T, Kanai R, Walsh V (2010) Modulating neuronal activity produces specific and long-lasting changes in numerical competence. Curr Biol 20: 2016–2020. 21055945 10.1016/j.cub.2010.10.007 PMC2990865

[61] Taschereau-Dumouchel V, Rossion B, Schyns PG, Gosselin F (2010) Interattribute Distances do not Represent the Identity of Real World Faces. Front Psychol 1: 159. 21833225 10.3389/fpsyg.2010.00159 PMC3153774

[62] Willenbockel V, Sadr J, Fiset D, Horne GO, Gosselin F, et al (2010) Controlling low-level image properties: the SHINE toolbox. Behav Res Methods 42: 671–684. 20805589 10.3758/BRM.42.3.671

[63] Brunoni AR, Amadera J, Berbel B, Volz MS, Rizzerio BG, et al (2011) A systematic review on reporting and assessment of adverse effects associated with transcranial direct current stimulation. Int J Neuropsychopharmacol 14: 1133–1145. 21320389 10.1017/S1461145710001690

[64] Nitsche MA, Paulus W (2001) Sustained excitability elevations induced by transcranial DC motor cortex stimulation in humans. Neurology 57: 1899–1901. 11723286 10.1212/wnl.57.10.1899

[65] Gandiga P, Hummel F, Cohen L (2006) Transcranial DC stimulation (tDCS): a tool for double-blind sham-controlled studies in brain stimulation. Clin Neurophysiol: 845–850. 10.1016/j.clinph.2005.12.003 16427357

[66] Faria P, Hallett M, Miranda PC (2011) A finite element analysis of the effect of electrode area and inter-electrode distance on the spatial distribution of the current density in tDCS. J Neural Eng 8: 066017. 22086257 10.1088/1741-2560/8/6/066017 PMC3411515

[67] Diekelmann S, Born J (2010) The memory function of sleep. Nat Rev Neurosci 11: 114–126. 20046194 10.1038/nrn2762

[68] Backhaus J, Junghanns K, Born J, Hohaus K, Faasch F, et al (2006) Impaired declarative memory consolidation during sleep in patients with primary insomnia: Influence of sleep architecture and nocturnal cortisol release. Biol Psychiatry 60: 1324–1330. 16876140 10.1016/j.biopsych.2006.03.051

[69] Massicotte-Marquez J, Decary A, Gagnon JF, Vendette M, Mathieu A, et al (2008) Executive dysfunction and memory impairment in idiopathic REM sleep behavior disorder. Neurology 70: 1250–1257. 18216303 10.1212/01.wnl.0000286943.79593.a6

[70] Buysse D, Reynolds C, Monk T, Berman S, Kupfer D (1989) The Pittsburgh Sleep Quality Index (PSQI): A new instrument for psychiatric research and practice. Psychiatry Res 28: 193–213. 2748771 10.1016/0165-1781(89)90047-4

[71] Gratton G, Coles M, Donchin E (1983) A new method for off-line removal of ocular artifact. Electroencephalogr Clin Neurophysiol 55: 468–484. 6187540 10.1016/0013-4694(83)90135-9

[72] Mercure E, Cohen Kadosh K, Johnson MH (2011) The n170 shows differential repetition effects for faces, objects, and orthographic stimuli. Front Hum Neurosci 5: 6. 21283529 10.3389/fnhum.2011.00006 PMC3031024

[73] Kanwisher N, Yovel G (2006) The fusiform face area: a cortical region specialized for the perception of faces. Philos Trans R Soc Lond B Biol Sci 361: 2109–2128. 17118927 10.1098/rstb.2006.1934 PMC1857737

[74] Rossion B, Joyce CA, Cottrell GW, Tarr MJ (2003) Early lateralization and orientation tuning for face, word, and object processing in the visual cortex. Neuroimage 20: 1609–1624. 14642472 10.1016/j.neuroimage.2003.07.010

[75] Rossion B, Delvenne JF, Debatisse D, Goffaux V, Bruyer R, et al (1999) Spatio-temporal localization of the face inversion effect: an event-related potentials study. Biol Psychol 50: 173–189. 10461804 10.1016/s0301-0511(99)00013-7

[76] Sadeh B, Yovel G (2010) Why is the N170 enhanced for inverted faces? An ERP competition experiment. Neuroimage 53: 782–789. 20558303 10.1016/j.neuroimage.2010.06.029

[77] Morgan HM, Klein C, Boehm SG, Shapiro KL, Linden DEJ (2008) Working memory load for faces modulates P300, N170, and N250r. J Cogn Neurosci 20: 989–1002. 18211245 10.1162/jocn.2008.20072 PMC2577178

[78] Fabiani M, Karis D, Donchin E (1986) P300 and recall in an incidental memory paradigm. Psychophysiology 23: 298–308. 3749410 10.1111/j.1469-8986.1986.tb00636.x

[79] Fabiani M, Karis D, Donchin E (1990) Effects of mnemonic strategy manipulation in a Von Restorff paradigm. Electroencephalogr Clin Neurophysiol 75: 22–35. 1688770 10.1016/0013-4694(90)90149-e

[80] Kim KH, Kim JH, Yoon J, Jung KY (2008) Influence of task difficulty on the features of event-related potential during visual oddball task. Neurosci Lett 445: 179–183. 18790010 10.1016/j.neulet.2008.09.004

[81] Antal A, Varga ET, Kincses TZ, Nitsche MA, Paulus W (2004) Oscillatory brain activity and transcranial direct current stimulation in humans. Neuroreport 15: 1307–1310. 15167555 10.1097/01.wnr.0000127460.08361.84

[82] Hsu TY, Tseng LY, Yu JX, Kuo WJ, Hung DL, et al (2011) Modulating inhibitory control with direct current stimulation of the superior medial frontal cortex. Neuroimage 56: 2249–2257. 21459149 10.1016/j.neuroimage.2011.03.059

[83] Luo W, Feng W, He W, Wang NY, Luo YJ (2010) Three stages of facial expression processing: ERP study with rapid serial visual presentation. Neuroimage 49: 1857–1867. 19770052 10.1016/j.neuroimage.2009.09.018 PMC3794431

[84] Rossion B, Jacques C (2008) Does physical interstimulus variance account for early electrophysiological face sensitive responses in the human brain? Ten lessons on the N170. Neuroimage 39: 1959–1979. 18055223 10.1016/j.neuroimage.2007.10.011

[85] Dering B, Martin CD, Moro S, Pegna AJ, Thierry G (2011) Face-sensitive processes one hundred milliseconds after picture onset. Front Hum Neurosci 5: 93. 21954382 10.3389/fnhum.2011.00093 PMC3173839

[86] Flevaris AV, Robertson LC, Bentin S (2008) Using spatial frequency scales for processing face features and face configuration: an ERP analysis. Brain Res 1194: 100–109. 18190897 10.1016/j.brainres.2007.11.071

[87] Pourtois G, Dan ES, Grandjean D, Sander D, Vuilleumier P (2005) Enhanced extrastriate visual response to bandpass spatial frequency filtered fearful faces: time course and topographic evoked-potentials mapping. Hum Brain Mapp 26: 65–79. 15954123 10.1002/hbm.20130 PMC6871777

[88] Kovacs G, Zimmer M, Banko E, Harza I, Antal A, et al (2006) Electrophysiological correlates of visual adaptation to faces and body parts in humans. Cereb Cortex 16: 742–753. 16120795 10.1093/cercor/bhj020

